# Anisotropic velocity properties and lithofacies identification of shahejie lacustrine shales

**DOI:** 10.1038/s41598-026-49001-4

**Published:** 2026-04-20

**Authors:** Min Li, Weihua Liu, Yang Wang, Junsheng Zhao, Hui Shen, Wenhui Tan

**Affiliations:** 1 Geophysical Laboratory, SINOPEC Geophysical Research Institute Co., Ltd., Nanjing, 211103 China; 2 SINOPEC Key Laboratory of Geophysics, Nanjing, 211103 China; 3 Research Institute of Geophysical Prospecting, SINOPEC Zhongyuan Oilfield Company, Puyang, 457001 China

**Keywords:** Energy science and technology, Solid Earth sciences

## Abstract

Lacustrine shales have gained increasing attention in the global energy transition. However, their more complex lithofacies variations compared to marine shales pose significant challenges for precise reservoir characterization. This study investigates the anisotropic elastic properties of Shahejie lacustrine shales to identify key parameters for lithofacies discrimination. Anisotropic ultrasonic velocity measurements were performed on 59 sample pairs, classified into four lithofacies, as argillaceous, mixed, siliceous, and calcareous. The results demonstrate distinct velocity and anisotropy behaviors across lithofacies, primarily governed by the alignment of clay particles, laminated kerogen, bedding planes, and associated micro-cracks. Although global linear P- and S-wave velocity trends and exponential relationships between bedding-normal velocities and Thomsen’s anisotropy parameters are observed, the bedding-normal P-wave velocity and P-to-S wave velocity ratio exhibit systematic, lithofacies-dependent variations. Consequently, a cross-plot template based on bedding-normal P-wave impedance and velocity ratio is established to effectively distinguish the four lithofacies. These findings provide critical rock-physics constraints for lithofacies prediction in Shahejie shales, supporting the optimization of exploration targets and development strategies in analogous lacustrine shale reservoirs.

## Introduction

Amidst the global energy transition and breakthroughs in unconventional hydrocarbon exploration, shale reservoirs have been at the forefront of petroleum geology research as critical source rocks and caprocks^1^. Lacustrine shale reservoirs, characterized by unique depositional settings and resource potential, have attracted significant attention. Some stratigraphic units such as the Shahejie Formation in the Bohaiwan Basin, the Qingshankou Formation in the Songliao Basin, and the Yangye Formation in the Tarim Basin demonstrate substantial exploration prospects^2–4^.Unlike marine shales, lacustrine shales deposit in restricted basins under aridity-humidity-alternating paleoclimatic cycles, high-frequency lake-level fluctuations, and multi-source sediment inputs. Hence, form thin interbeds, rapid lithological changes, and heterogeneity depositional sequences^5^.Vertically, complex mineralogical variations of millimeter-scale laminations create multi-lithofacies compositions. And the spatial differentiation of lithofacies directly governs the capacity, quality, frackability, and fluid mobility of shale reservoirs, making precise lithofacies identification essential for optimizing exploration targets and mitigating development risks.

Lithofacies, defined as rock units with distinct mineral assemblages, sedimentary structures, and genetic significance, encapsulate multiscale controls of depositional-diagenetic processes on reservoir properties. In shale hydrocarbon exploration, the strong correlation between lithofacies and reservoir performance has driven the development of ternary classification frameworks according to mineral composition, bedding architecture, and total organic carbon (TOC)^6–8^. And the rock physical parameters vary markedly across lithofacies, the experimental research of Liu et al. on Jurassic lacustrine shales shows that calcareous shales have high elastic moduli and weak anisotropy, whereas argillaceous shales display strong anisotropy and low stiffness^9^. Zhang et al. revealed the differences in elastic wave velocity and modulus among three main lithofacies in the Permian Wujiaping Formation^10^. Such rock physical characteristics are transformed into identifiable geophysical signals through seismic wavefield propagation effects (e.g. AVO, anisotropic attenuation), underscoring the need for rock physics-driven lithofacies identification to seismic ‘sweet spot’ prediction.

Shales, as typical vertical transverse isotropy (VTI) media, exhibit elastic anisotropy governed by five independent stiffness coefficients based on Hooke’s law^11^. Thomsen simplified anisotropy quantification via three dimensionless parameters $\varepsilon$, $\gamma$ and $\delta$^12^. Laboratory studies attribute shale anisotropy to laminated clay alignment, lenticular kerogen distribution, and bedding-parallel microfractures^13–20^. Despite consensus on these mechanisms, the lithofacies-dependent modulation of anisotropy remains poorly understood.

Due to the regional specificity of rock physical understanding, which cannot be arbitrarily generalized, studies on rock physics and lithofacies identification in specific research areas hold significant scientific value. This study focuses on the lacustrine shale of the Shahejie Formation. By rock compositional and rock physical experimental tests, we first establish a lithofacies classification scheme and summarize their mineralogical composition and microstructural characteristics. Subsequently, we reveal the confining pressure-dependent behavior of elastic parameters for different lithofacies and their dominant control factors. Finally, a $v_P-v_S$ linear empirical relationship and lithofacies identification cross-plot with threshold delineation are developed, providing a rock physical solution for lithofacies prediction of Shahejie shales in the Bohaiwan Basin.

## Geological setting

The Dongpu Depression is situated in the southern sector of the Bohaiwan Basin, China, with a general NNE orientation. It is a petroliferous Cenozoic faulted basin characterized by lacustrine saline lake deposits^2^. Its structural framework, shaped by the Yanshan and Himalayan orogenic movements, can be described as ‘two sags, one uplift, one steep slope, and one gentle slope’. The Cenozoic stratigraphic sequence comprises, from oldest to youngest, the Paleogene Shahejie Formation (Es) and Dongying Formation (Ed), followed by the Neogene Guantao Formation (Ng) and Minghuazhen Formation (Nm), and the Quaternary Pingyuan Formation (Qp).

**Fig. 1:**
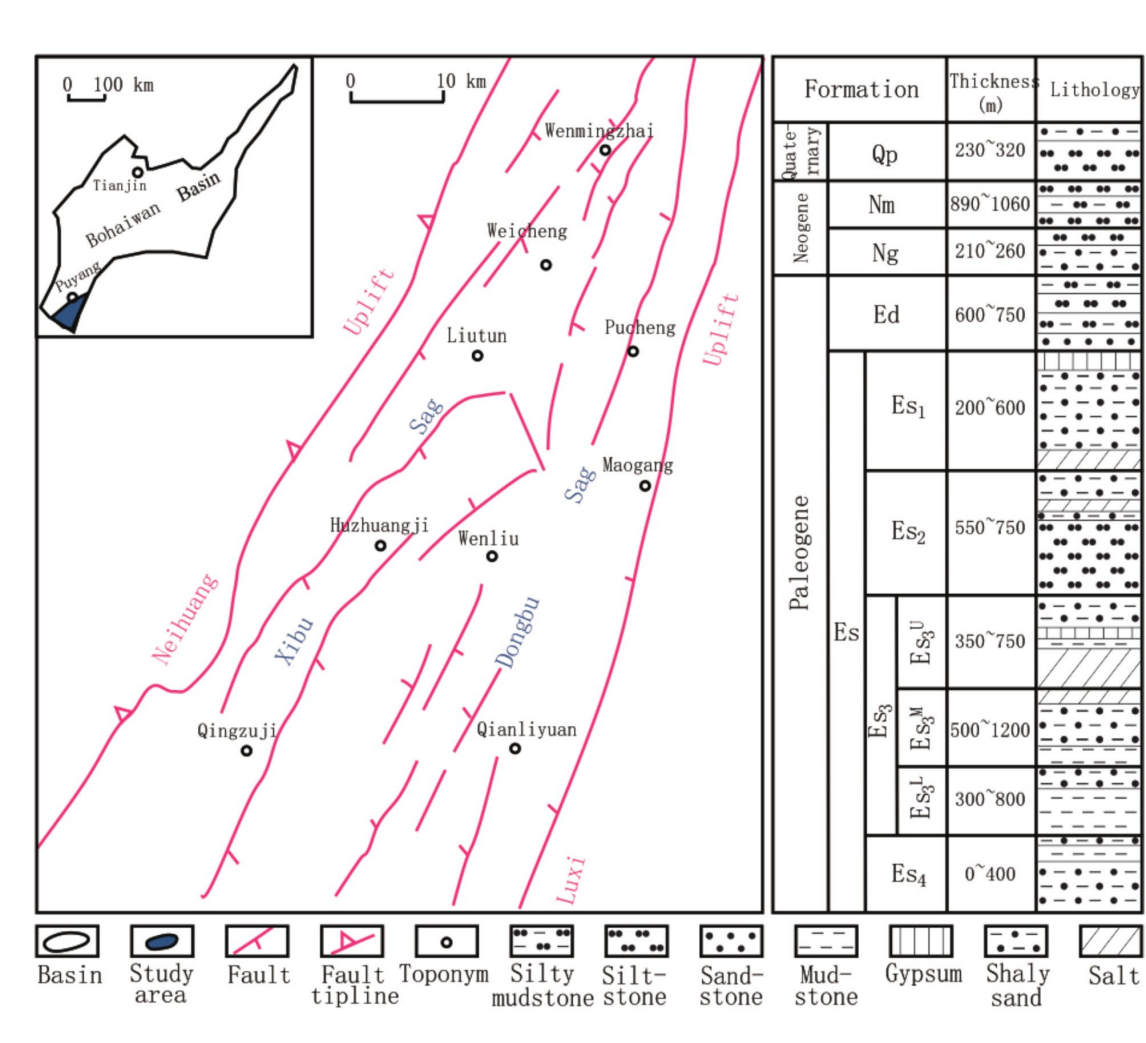
Structural Outline of Dongpu Depression in Bohaiwan Basin and comprehensive stratigraphic column of Cenozoic strata.^23^.

The Shahejie Formation is the critical stratum for hydrocarbon source rock development and the primary target for exploration in the depression. With a considerable total thickness ranging from 2,000 to 5,000 meters, it is subdivided into four members, i.e., Es1, Es2, Es3, and Es4 (from top to bottom). The depositional environment during this period was complex, influenced by frequent alternations between arid and humid climates coupled with significant lake-level fluctuations. This resulted in a diverse assemblage of lithologies, including terrigenous clastics, carbonates, salt, and gypsum, which interbed to form multiple sedimentary rhythms^21,22^ (Fig. 1).

## Materials and methods

### Sample description and laboratory measurements

To conduct a comprehensive study of rock physics properties of lacustrine shales with different lithofacies, we collect 59 full-diameter downhole core samples from Dongpu Depression. The collected shale samples belong to the Shahejie Formation of the Paleogene system, with the burial depth ranging from 3682 m to 3968 m.

**Fig. 2:**
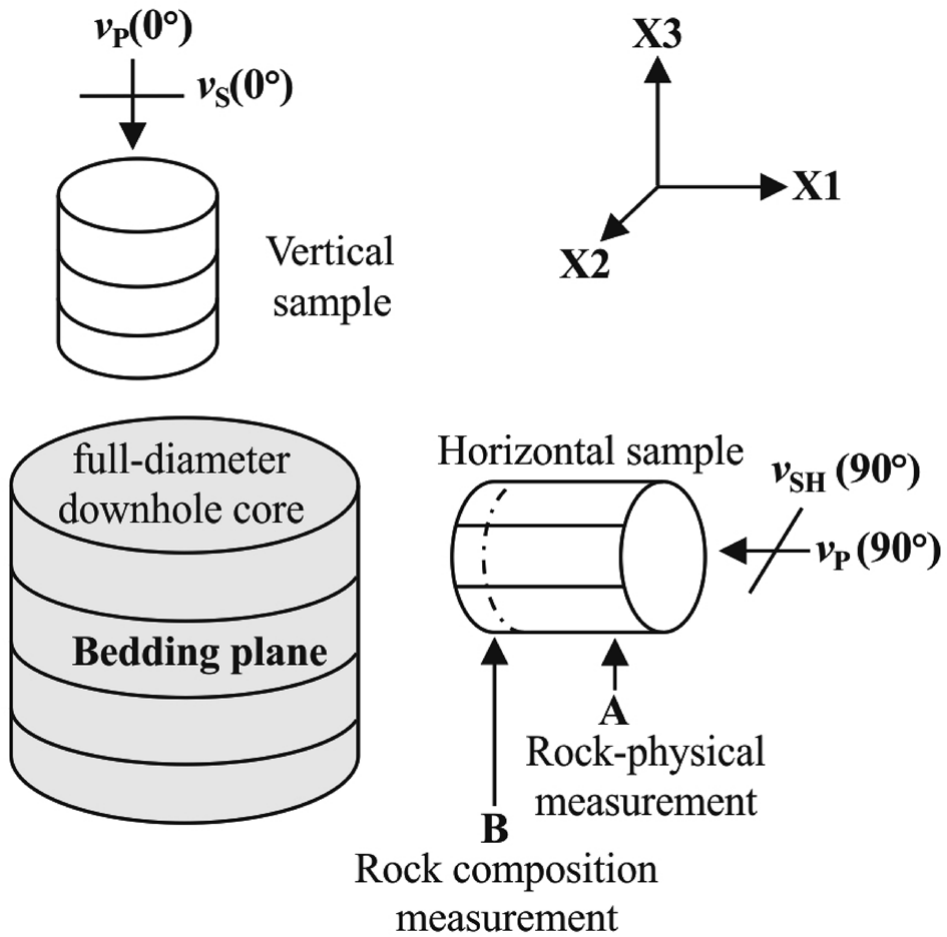
Principles of coring from a full-diameter sample with a transversely isotropic structure. In the X1/X2/X3 coordinate system, velocities measured on vertical and horizontal samples are shown. Portion B is used for rock composition and thin-section measurement^9,27^.

Figure 2 schematically illustrates the sample preparation procedure. A pair of cylindrical shale samples, perpendicular and parallel to the bedding plane, are cut off from each downhole core using the wire-cutting apparatus^24^. The cylinders are ensured to have a diameter of 2.5 cm and a length-to-diameter ratio of 1.5-2.0^25^. All prepared samples are placed at an electric oven at the temperature of $80^{\circ }$C for over 48 hours to achieve thorough drying. Following drying, the bulk density of each dried cylinder is measured by the weight and the dimension. Porosity is measured according to the Boyle’s law by using the Ultra-Pore 300 porosimeter. Ultrasonic velocities are measured by using a pulse transmission method^26^. In this setup, the cylindrical sample, encased in a rubber sleeve, was placed between two endcaps equipped with piezoelectric transducers (1 MHz dominant frequency) and subjected to confining pressure within a silicon oil-filled vessel. Measurements were taken at confining pressures from 5 to 55 MPa in 10 MPa increments, with an accuracy of ±1% for P-wave and ±2% for S-wave velocities. Additionally, the end-fragment of the horizontal sample (Section B in Fig. 2) in the cutting process are used to conduct the X-ray powder diffraction (XRD) tests and the rock-eval pyrolysis tests to gain the mineral composition and the total organic carbon (TOC). The thin section analysis are also performed with the end-fragment.

### Lithofacies classification based on mineralogy

**Fig. 3:**
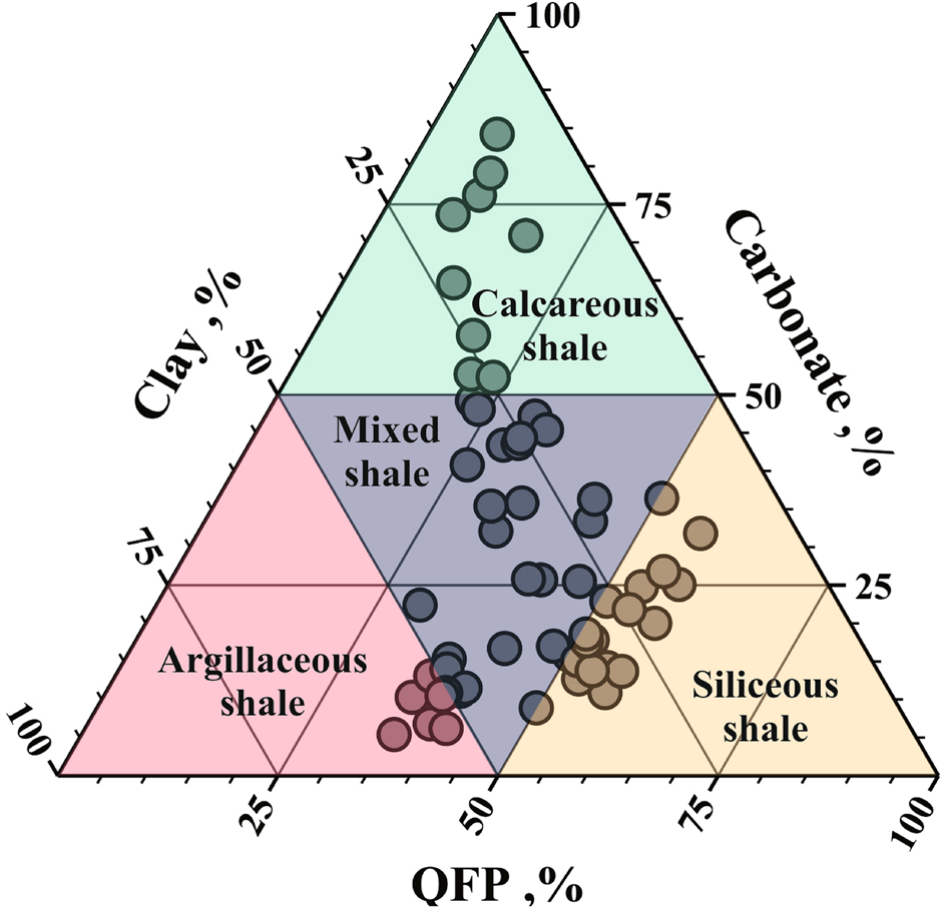
Ternary diagram of mineral compositions and lithofacies classification of Shahejie lacustrine shales.

The mineralogical composition of the 59 shale samples, as determined by XRD tests, is presented in the ternary diagram of Fig. 3, where the vertices represent quartz-feldspar-pyrite (QFP), clay, and carbonate mineral groups. A quantitative classification scheme was applied based on the dominant mineral constituent. When a sample is assigned to a specific lithofacies when the content of any single mineral group (QFP, clay, or carbonate) exceeds 50% of the total composition, and samples that do not meet this dominance threshold for any individual group are classified as ‘Mixed’ lithofacies. Accordingly, the samples are categorized into four distinct types as Argillaceous (clay> 50%), Siliceous (QFP> 50%), Calcareous (carbonate> 50%), and Mixed (no single group > 50%)^9^.

### Calculation of anisotropy parameters

From paired cylinders, as shown in Fig. 2, we obtain two P-wave velocities ($v_P(0^\circ )$, $v_P(90^\circ )$) and two S-wave velocities ($v_S(0^\circ )$, $v_{SH}(90^\circ )$). As $v_S(0^\circ )$ denotes S-wave propagating in bedding-normal direction but vibrating in bedding-parallel direction, while $v_{SH}(90^\circ )$ indicates S-wave propagating and vibrating both in bedding-parallel direction.

Laboratory characterization of velocity anisotropy for unconventional shales is commonly performed under the assumption of transverse isotropy (TI), wherein the elastic behavior can be fully described by five independent stiffness parameters: $C_{11}$, $C_{33}$, $C_{44}$, $C_{66}$, and $C_{13}$^1,11,13^. With the measured ultrasonic velocities and bulk density ($\rho$), four stiffness parameters can be derived:

1 $$\begin{aligned} & &{C_{11} =\rho v_P^2(90^\circ )} \end{aligned}$$

2 $$\begin{aligned} & &{C_{33} =\rho v_P^2(0^\circ )} \end{aligned}$$

3 $$\begin{aligned} & &{C_{44} =\rho v_S^2(0^\circ )} \end{aligned}$$

4 $$\begin{aligned} & &{C_{66} =\rho v_{SH}^2(90^\circ )} \end{aligned}$$

Subsequently, P- and S-wave velocity anisotropy can be quantified with Thomsen’s dimensionless parameters^12^, $\epsilon$ and $\gamma$, as follows:

5 $$\begin{aligned} & &\epsilon = \frac{C_{11}-C_{33}}{2C_{33}} \end{aligned}$$

6 $$\begin{aligned} & &\gamma = \frac{C_{66}-C_{44}}{2C_{44}} \end{aligned}$$

## Results

### Microstructural, compositional and pore properties

**Fig. 4:**
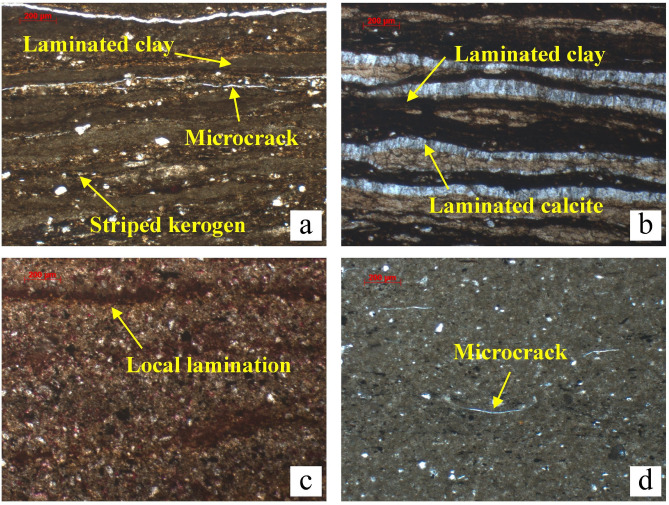
Thin section images for (**a**) argillaceous shale, (**b**) mixed shale, (**c**) siliceous shale, and (**d**) calcareous shale.

Figure 4 displays thin section images for four typical samples with (a) argillaceous, (b) mixed, (c) siliceous, and (d) calcareous lithofacies, respectively. Shahejie shales with the burial depth beyond 3500 m have undergone the smectite-to-illite transition^28^, resulting in clay minerals mainly composing of illite and illite/smectite mixed layers. Clay minerals show obvious direction arrangement, forming individual lamina, especially for argillaceous and mixed shales (Fig.4a and b). Additionally, in argillaceous and mixed shales, kerogen in the middle stage of maturity (Ro$\approx$1.0%) prefers to develop along the laminated clay with a stripped pattern. The laminated clay, micritic carbonate, and kerogen exhibit a ribbon-like morphology. The siliceous and calcareous shales in Fig.4c and d overall display a massive structure. The siliceous shale mainly consists of mud-to-silt quartz and feldspar with local lamination, while the calcareous shale is predominantly composed of micritic-to-micro-crystalline dolomite. Kerogen in these two lithofacies distributes with a sporadic pattern.

**Fig. 5:**
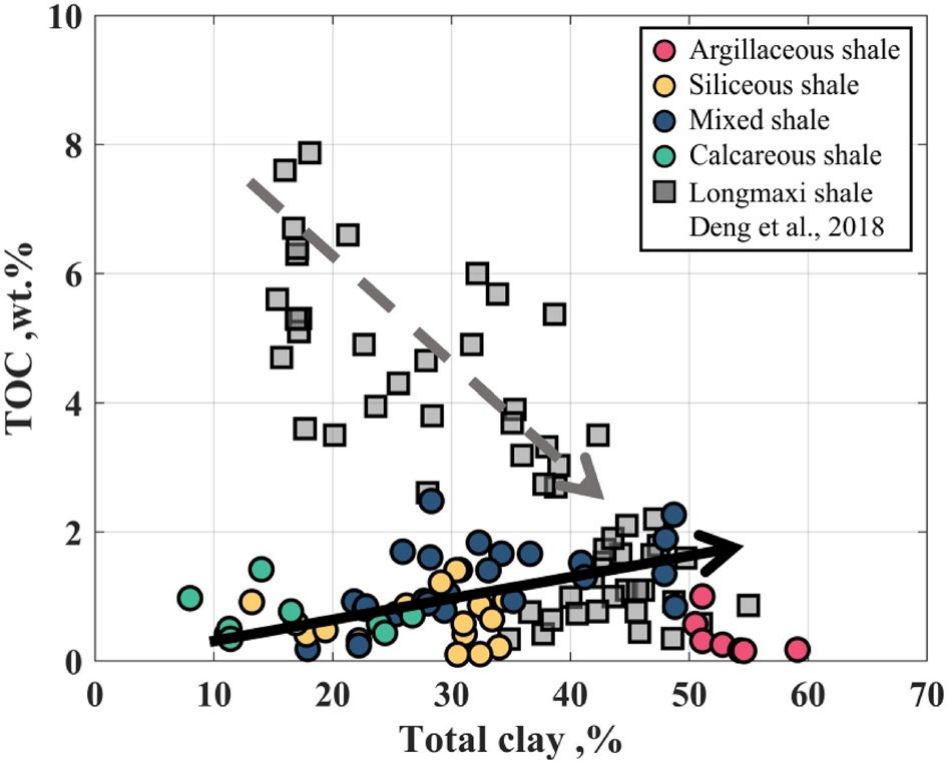
TOC as a function of the total clay content for four lithofacies identified in Shahejie shales. For comparison, data from the Longmaxi marine shales in the Sichuan Basin, China^29^ are also included.

Figure 5 plots TOC against the total clay content for four types of lithofacies identified in Shahejie shales. Overall, the TOC content of Shahejie shales is distributed between 0% to 2.5%. In contrast to Longmaxi marine shales^29^, Shahejie shales exhibit a broader range of clay content and significantly lower TOC, highlighting one of the fundamental differences between lacustrine and marine shale sedimentary systems. There exists a positive clay-TOC correlation for Shahejie shales, and TOC of calcareous, siliceous, and argillaceous shales is generally less than 1.5%, while that of mixed shales can reach up to 2.5%, suggesting that clay minerals are favorable for organic matter enrichment. In contrast, the Longmaxi marine shales show a negative clay-TOC correlation, because organic matter has a biogenic siliceous origin^29^.

**Fig. 6:**
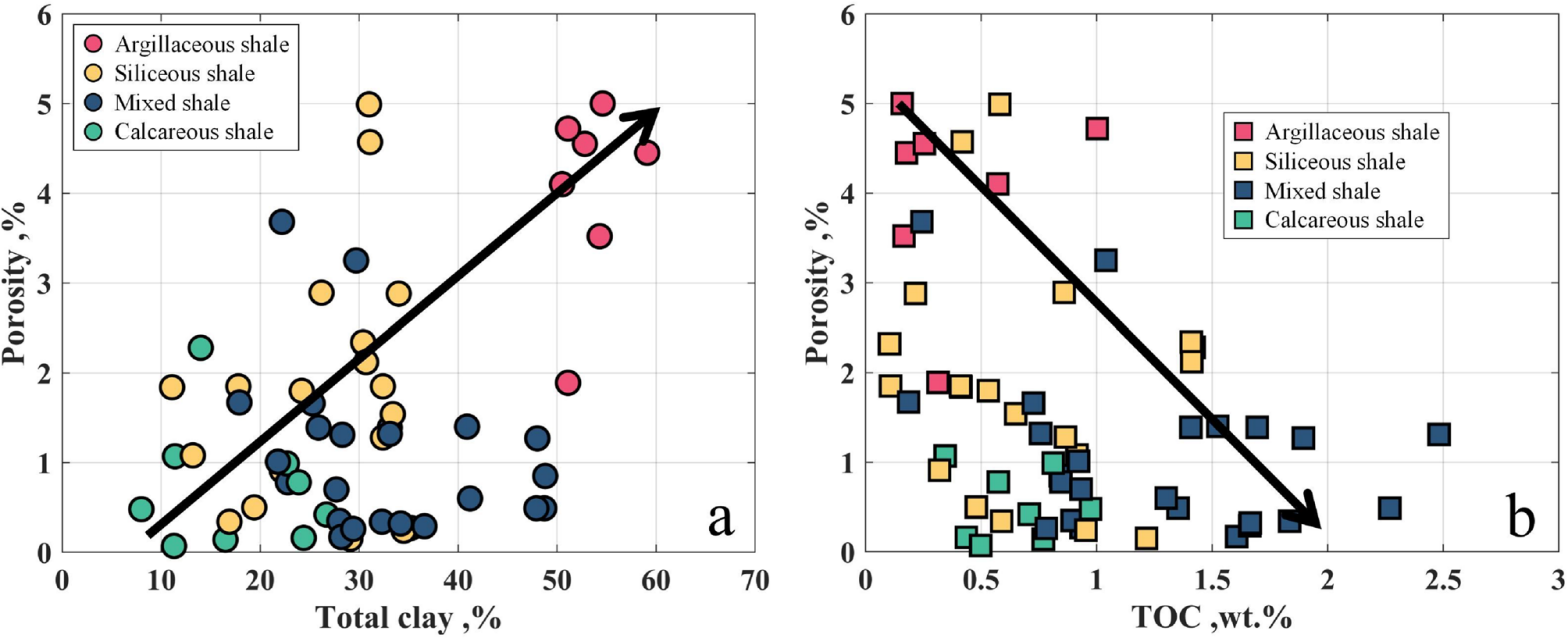
Porosity as a function of (**a**) the total clay content and (**b**) TOC for four lithofacies of Shahejie shales.

Figure 6 displays the porosity as a function of (a) the total clay content and (b) TOC. Porosity distributes between 0% and 5%. The positive correlation between porosity and total clay content, coupled with the negative correlation between porosity and TOC, to some extent, reveals the predominance of inorganic pores and the underdevelopment of organic-related pores in Shahejie shales. The different porosity ranges among lithofacies reveal differences in pore structure. Specifically, argillaceous shales have the highest porosity (ranging from 2% to 5%, with an average of 4.03%), followed by siliceous (0–5%, 1.87%), mixed (0–4%, 1.05%), and calcareous shales (0–2.5%, 0.71%). This indicates that micro-cracks generated in the process of smectite-to-illite transition might be an important contributor for inorganic pores, while the process of carbonate cementation might restrain the development of inorganic pore spaces^28^.

### Pressure-dependent properties of different lithofacies

**Fig. 7:**
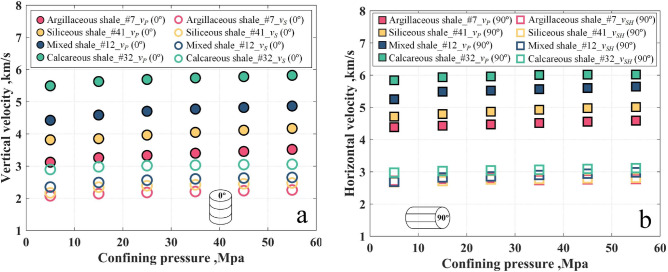
P- and S-wave velocities as a function of the applied confining pressure for four lithofacies in (**a**) vertical and (**b**) horizontal directions.

Figure 7 displays (a) vertical velocities and (b) horizontal velocities as a function of the applied confining pressure for four samples representing the argillaceous, siliceous, mixed, and calcareous lithofacies. From the whole, all velocities show increasing trends over the entire pressure range from 5 to 55 MPa. Vertical velocities display more pressure sensitivity than horizontal velocities, which might be attributed to the progressive closure of bedding-parallel micro-cracks with elevated confining pressure. Furthermore, both P-wave and S-wave velocities follow a magnitude relationship as: calcareous >mixed >siliceous >argillaceous, reflecting the inherent stiffness contrast among terminal minerals. Notably, P-wave velocity differences among four lithofacies far exceed those of S-waves. In the vertical direction, the P-wave velocity disparity ($v_P(0^\circ )$) between calcareous and argillaceous shales reaches approximately 2.5 km/s, compared to an S-wave difference ($v_S(0^\circ )$) of 0.9 km/s, highlighting significant velocity ratio variations across lithofacies. In the horizontal direction, the P-wave velocity difference ($v_P(90^\circ )$) reduces to 1.5 km/s, while S-wave velocity difference ($v_{SH}(90^\circ )$) narrows to 0.4 km/s between calcareous and argillaceous shales. Additionally, for both P- and S-waves, horizontal velocities surpass vertical velocities for all samples, exhibiting obvious anisotropy. The anisotropy degree orders as calcareous >mixed >siliceous >argillaceous shales, demonstrating lithofacies-dependent properties.

**Fig. 8:**
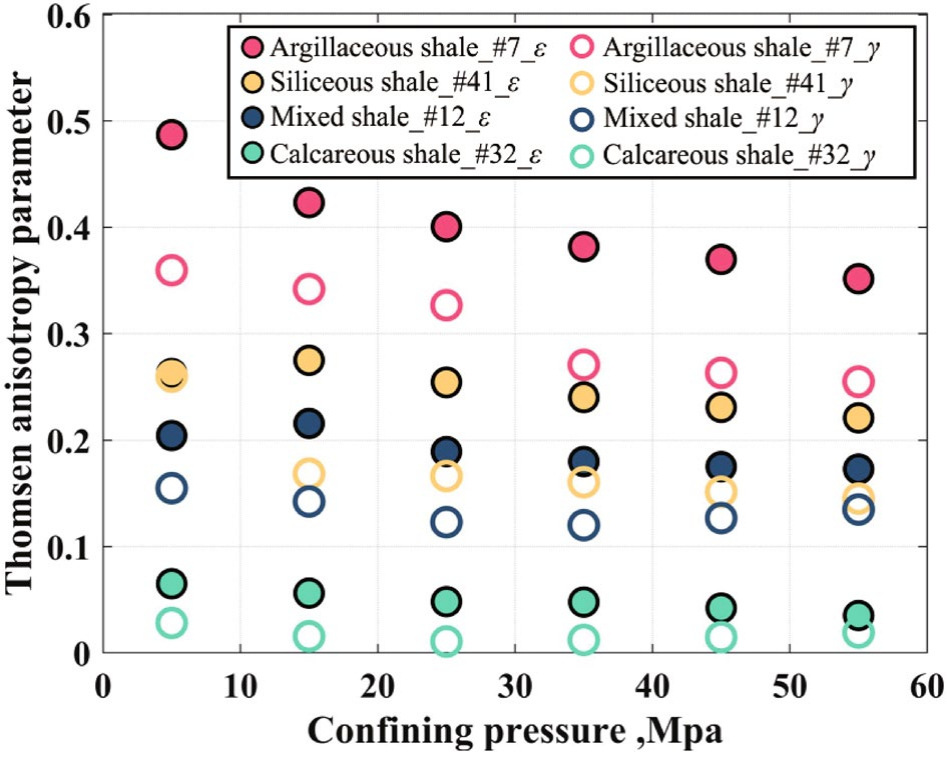
Thomsen’s anisotropy parameters $\epsilon$ and $\gamma$ as a function of the confining pressure for four lithofacies.

P- and S-wave velocity anisotropy are quantitatively expressed with Thomsen’s parameters $\epsilon$ and $\gamma$. Figure 8 plots $\epsilon$ and $\gamma$ against the applied confining pressure. Overall, the Shahejie shales exhibit moderate anisotropy with parameter values below 0.5. As the confining pressure increases from 5 to 55 MPa, both $\epsilon$ and $\gamma$ progressively decrease to their intrinsic values. The pressure-dependent anisotropy, to some extent, could be attributed to the closure of micro-cracks subparallel to beddings. Additionally, both $\epsilon$ and $\gamma$ differ with lithologies, following a rough pattern: argillaceous >siliceous >mixed >calcareous shales.

### Factors influencing velocity anisotropy

**Fig. 9:**
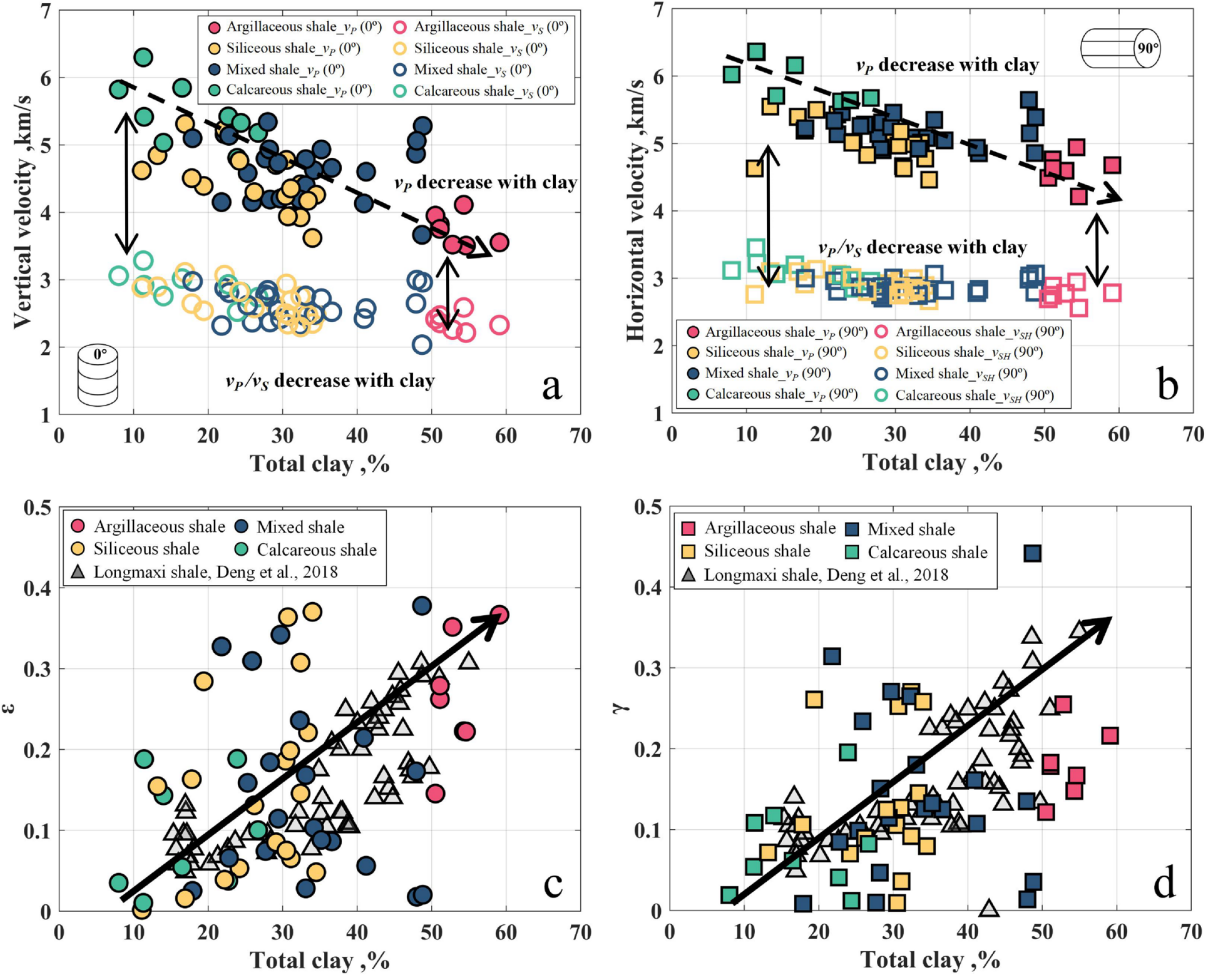
The relationships between total clay content and (**a**) vertical velocity, (**b**) horizontal velocity, (**c**) $\epsilon$ and (**d**) $\gamma$ of four lithofacies. For comparison, data from the Longmaxi marine shales in the Sichuan Basin, China^29^ are also included in (**c**) and (**d**).

**Fig. 10:**
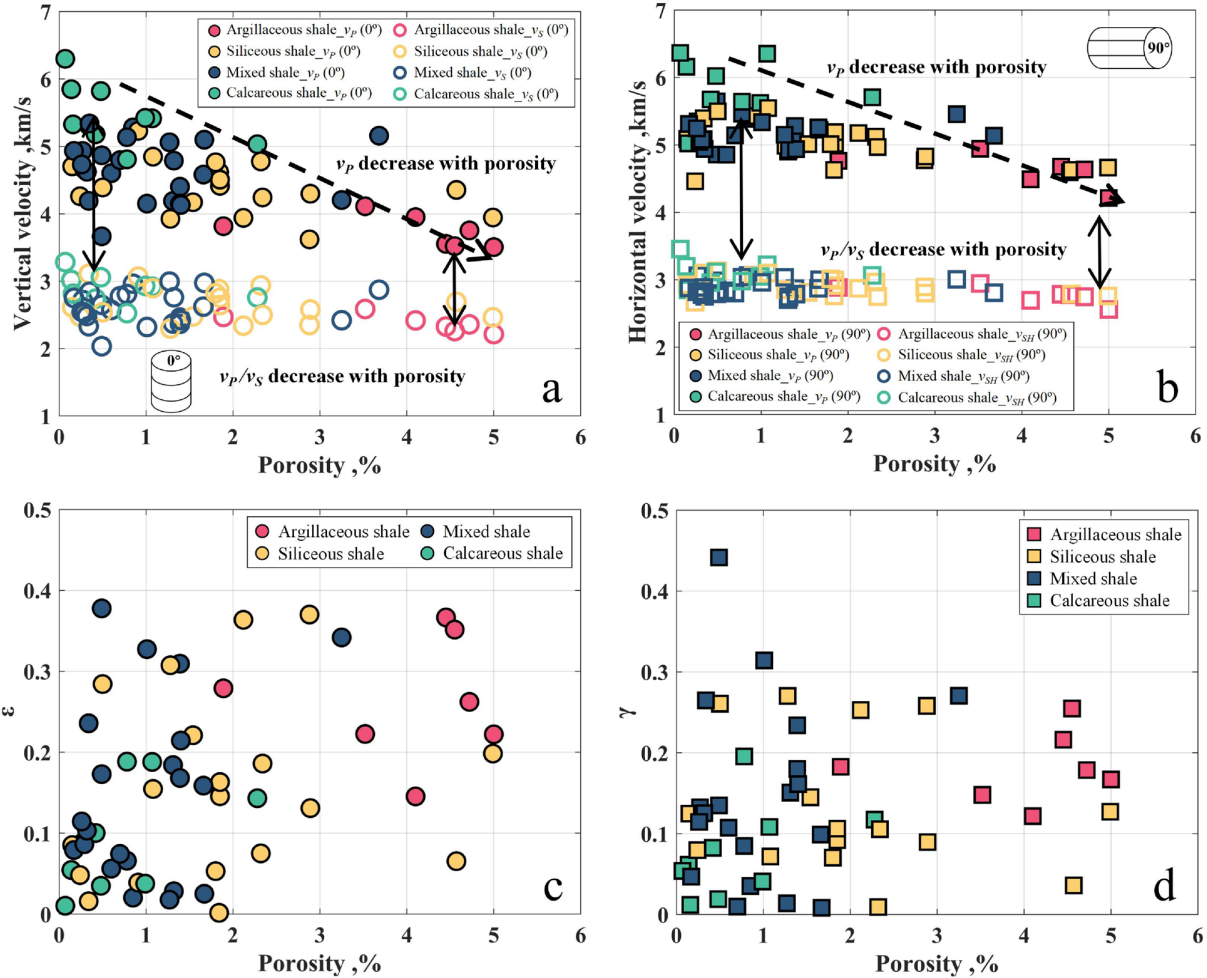
The relationships between porosity and (**a**) vertical velocity, (**b**) horizontal velocity, (**c**) $\epsilon$ and (**d**) $\gamma$ of four lithofacies.

**Fig. 11:**
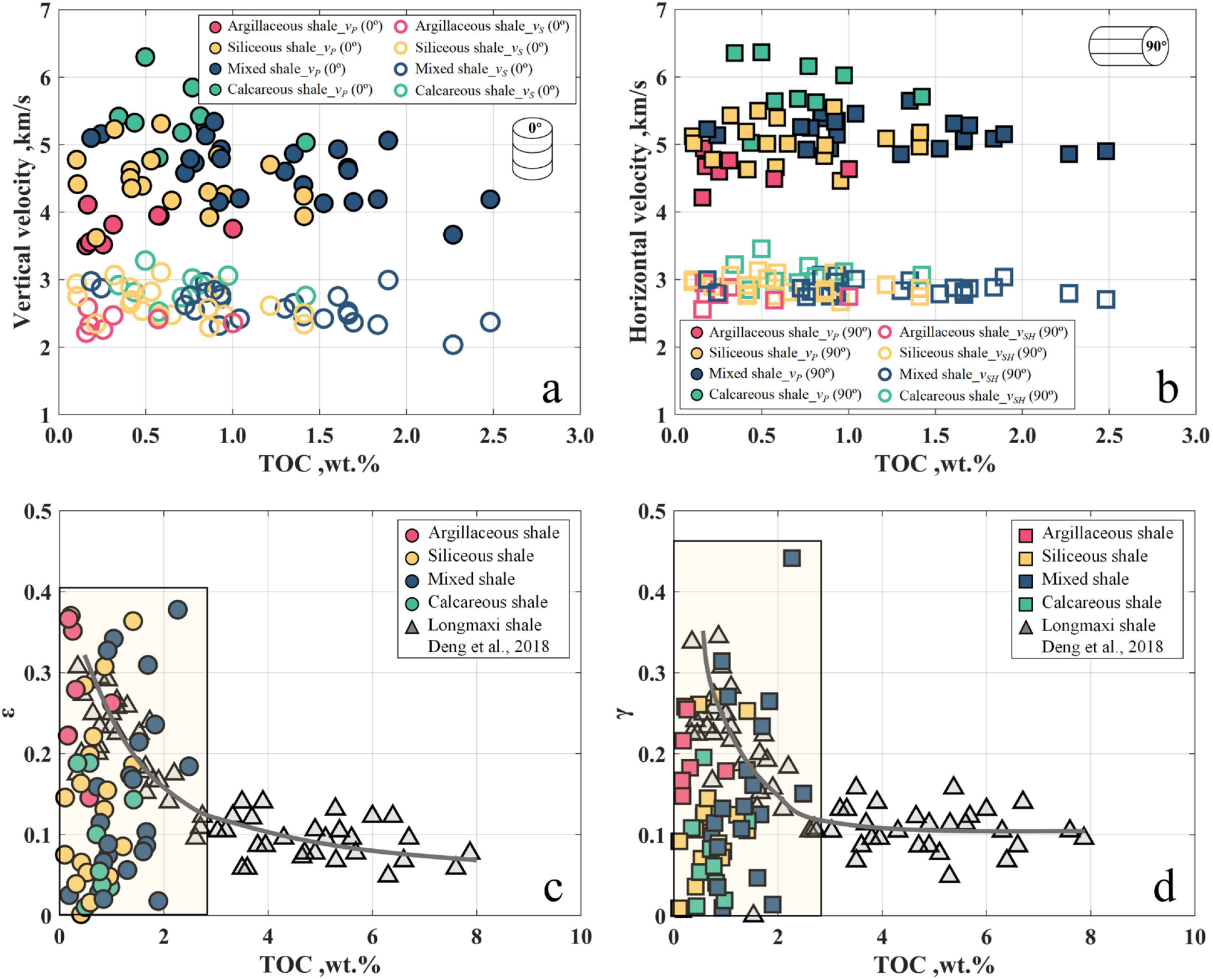
The relationships between TOC and (**a**) vertical velocity, (**b**) horizontal velocity, (**c**) $\epsilon$ and (**d**) $\gamma$ of four lithofacies. For comparison, data from the Longmaxi marine shales in the Sichuan Basin, China^29^ are also included in (**c**) and (**d**).

The influences of three key factors, namely total clay content, porosity, and TOC, on velocity and anisotropy are analyzed using experimental data at 55 MPa confining pressure (Figs.9, 10, 11). This pressure approximates the in-situ vertical effective stress in Shahejie reservoirs^30^, thereby revealing the intrinsic elastic variations dictated by compositional and structural^1^.

Figure 9 illustrates relationships between the total clay content and (a) vertical velocities, (b) horizontal velocities, (c) $\epsilon$ and (d) $\gamma$. For comparison, data from the Longmaxi marine shales in the Sichuan Basin^29^ are also included in parts (c) and (d). As shown in Fig. 9a and b, velocities generally decrease with the increasing clay content. When clay rises from 8% to 58%, $v_P(0^\circ )$ decreases from 6.3 to 3.5 km/s, while $v_S(0^\circ )$ decreases from 3.3 to 2.1 km/s, indicating high sensitivity of $v_P(0^\circ )$ and $v_P(0^\circ )/v_S(0^\circ )$ to terminal clay mineral variations, as indicated by the arrows. Similarly, $v_P(90^\circ )$ and $v_{SH}(90^\circ )$ also exhibit clay-dependent trends, albeit with reduced sensitivity compared to vertical velocities. The clay variation results in a $v_P(90^\circ )$ difference of 2.2 km/s and a $v_{SH}(90^\circ )$ difference of 0.9 km/s, approximately 30% smaller than vertical velocities. In Fig. 9c,d, both $\epsilon$ and $\gamma$ generally increase with the total clay content. Shahejie shales have moderate anisotropy of $\epsilon<$ 0.4 and $\gamma<$ 0.45. Argillaceous shales exhibit much stronger anisotropy in contrast to calcareous shales, indicating the oriented arrangement of clay minerals is the main controlling factor for velocity anisotropy. This is also confirmed by the thin section images in Fig.4. Additionally, compared to Longmaxi marine shales, Shahejie lacustrine shales have comparative anisotropy degree and demonstrate a more scattered anisotropy-clay relationship.

Figure 10 plots (a) vertical velocities, (b) horizontal velocities, (c) $\epsilon$ and (d) $\gamma$ as a function of porosity. As shown by the dashed arrows in Fig. 10a,b, both $v_P(0^\circ )$ and $v_P(90^\circ )$ decrease with increasing porosity, while $v_S(0^\circ )$ and $v_{SH}(0^\circ )$ remain almost unaffected. This results in a systematic reduction of the velocity ratio with porosity increment, as indicated by the two-way arrows. Although porosity distribution of four lithologies overlap, calcareous shales generally have the lowest porosity, followed by siliceous, mixed, and argillaceous shales. The porosity magnitude directly contributes to the observed differences in P-wave velocities and velocity ratios across lithofacies. In Fig. 10c,d, $\epsilon$ and $\gamma$ display weakly positive correlation with porosity, which might be caused by the micro-cracks among beddings in argillaceous and mixed shales^31^, as shown in Fig. 4a,b.

Figure 11 plots (a) vertical velocities, (b) horizontal velocities, (c) $\epsilon$ and (d) $\gamma$ against TOC. As shown in Fig. 11a,b, P- and S-wave velocities display very small variations over the entire TOC range (0–2.5%). However, $\epsilon$ and $\gamma$ (highlighted by boxes in Fig. 11c,d) display a positive correlation with TOC content, which might be attributed to the strip-like kerogen subparallel to beddings^8,19,32^, as indicated in Fig. 4. In contrast, for Longmaxi marine shales, anisotropy parameters gradually decrease with the increasing TOC. This discrepancy may stem from the dispersed distribution of over-mature kerogen derived from biogenic silica^14,17,29,33^, which modifies the stress-dependent anisotropy framework.

## Discussion

### Relationship between P- and S-wave velocities

Understanding relationships between P- and S-wave velocities in unconventional shales is fundamental for geophysical and geomechanical applications, such as predicting missing S-wave logs and deriving key elastic parameters for reservoir characterization^34,35^. While global compilations of shale data have established general $v_P-v_S$ trends, the influence of specific mineralogy and lithofacies in complex lacustrine systems remains less constrained. For instance, Qin et al.^34^ analyzed a global dataset and concluded that mineralogy has a minor effect on $v_P-v_S$ relationships in marine shales. However, this finding may not directly extend to lacustrine shales like those in the Shahejie Formation, where lithofacies and mineral composition exhibit greater heterogeneity. This study, therefore, focuses on evaluating the $v_P-v_S$ relationships within and across distinct lithofacies to assess their diagnostic potential.

**Fig. 12:**
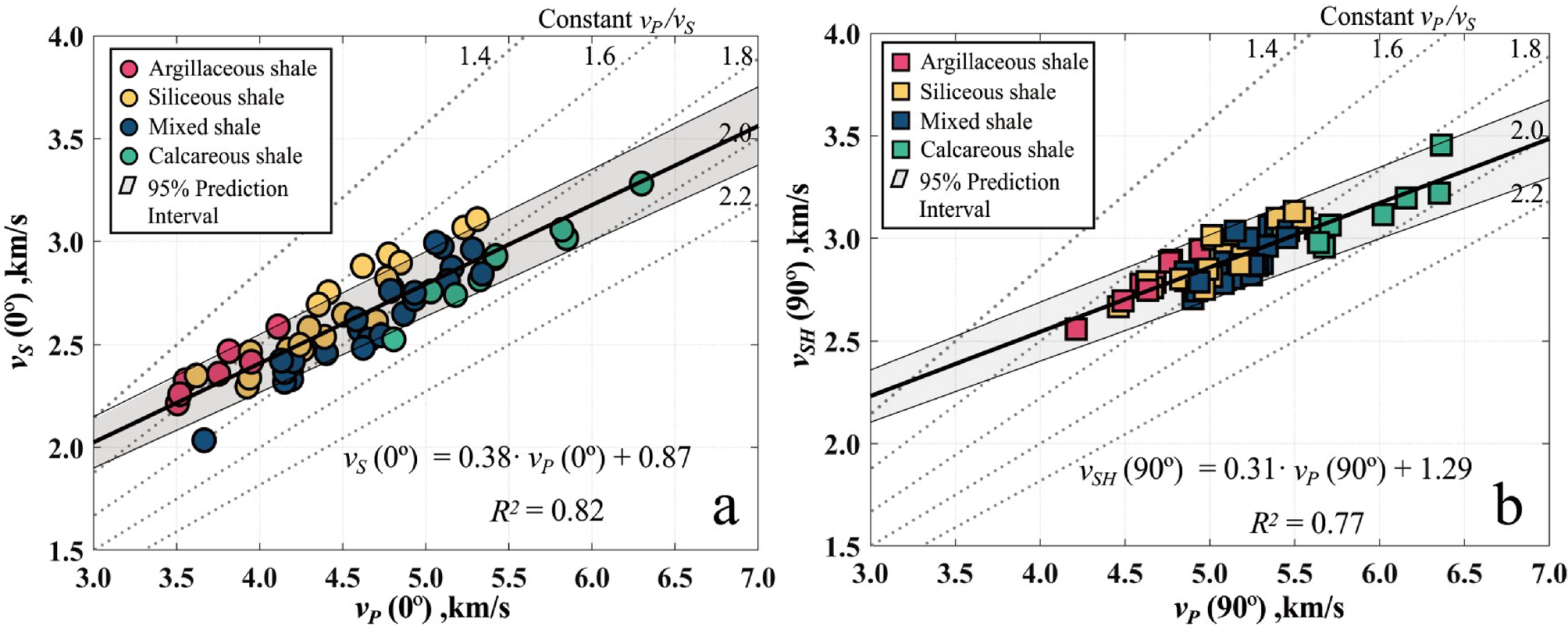
The cross-plot of $v_P-v_S$ relations for Shahejie lacustrine shale reservoirs in (**a**) vertical and (**b**) horizontal directions. The solid line in each panel represents the linear fit ($Y=a \cdot x+b$) with the coefficients of determination ($R^2$) texted in the figure. The grey areas mark the 95% prediction interval. The dotted lines are constant $v_P/v_S$ lines.

As shown in Fig. 12, $v_P$ is plotted as a function of $v_S$ for Shahejie lacustrine shales in the (a) vertical and (b) horizontal directions. The data reveal a strong first-order linear correlation in both directions, expressed as,

7 $$\begin{aligned} & &v_S(0^\circ ) = 0.38 \cdot v_P(0^\circ ) +0.87,\ R^2=0.82 \end{aligned}$$

8 $$\begin{aligned} & &v_{SH}(90^\circ ) = 0.31 \cdot v_P(90^\circ ) +1.29,\ R^2=0.77 \end{aligned}$$

The slightly stronger correlation in the vertical direction ($R^2$ = 0.82) compared to the horizontal ($R^2$ = 0.77), along with the lower average $v_P/v_S$ ratio vertically, likely reflects the anisotropic influence of bedding structures^36^, as shown in Fig. 4. More importantly, systematic lithofacies-controlled variations are evident despite the overall linear trend. Calcareous shales exhibit distinctly higher $v_P/v_S$ ratios (approximately 1.8–2.0) compared to argillaceous shales, while mixed and siliceous shales show overlapping values. Critically, the slopes of the best-fit lines for the vertical $v_P/v_S$ relationship vary by lithofacies. This observation indicates that the bedding-normal $v_P/v_S$ ratio is a more sensitive lithofacies discriminator than the horizontal ratio or the absolute velocities alone, providing a refined parameter for lithofacies classification in this complex lacustrine sequence.

### Relationship between velocities and anisotropy parameters

**Fig. 13:**
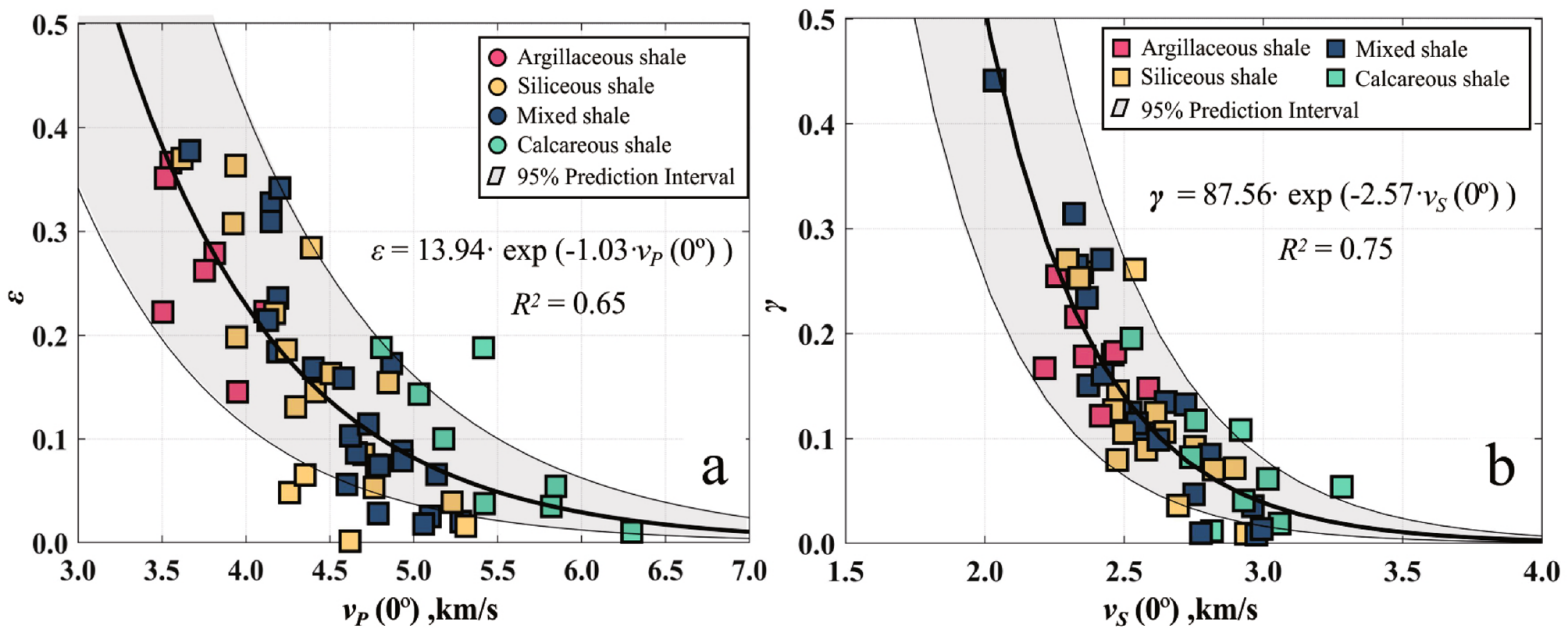
(**a**) The P-wave anisotropy $\epsilon$ versus the vertical P-wave velocity $v_P(0^\circ )$ and (**b**) the S-wave anisotropy $\gamma$ versus the vertical S-wave velocity $v_S(0^\circ )$. The solid line in each panel represents the linear fit ($Y=a \cdot exp(b \cdot x)$) with the coefficients of determination ($R^2$) texted in the figure. The grey areas mark the 95% prediction interval.

To facilitate anisotropy prediction for Shahejie shales, we establish two empirical models to straightforwardly predict Thomsen’s anisotropy parametes by plotting $\epsilon$ and $\gamma$ together with bedding-normal $v_P(0^\circ )$ and $v_S(0^\circ )$, respectively, as shown in Fig. 13. In general, both $\epsilon$ and $\gamma$ increase with the decreasing $v_P(0^\circ )$ and $v_S(0^\circ )$, which can be characterized by the following exponential relationships (with $R^2$ values of 0.65 and 0.75, respectively),

9 $$\begin{aligned} & &\epsilon = 13.94 \cdot exp (-1.03\cdot v_P(0^\circ )),\ R^2=0.65 \end{aligned}$$

10 $$\begin{aligned} & &\gamma = 87.56 \cdot exp (-2.57\cdot v_S(0^\circ )),\ R^2=0.75 \end{aligned}$$

It should be noted that the exponential relations are in accordance with many previous researches on unconventional shales^9,19,37^. However, for Shahejie shales with complex lithofacies in the current study, relations between vertical velocities and anisotropy parameters might differ with lithofacies, as listed in Table 1. The result shows that the distributions of anisotropy parameters among different lithofacies overlap significantly, making it difficult to effectively distinguish lithofacies. However, the P-wave velocity $v_P(0^\circ )$ can reflect lithological changes to some extent, roughly satisfying: argillaceous < mixed $\approx$ siliceous < calcareous. Both $v_P(0^\circ )$ and bulk density in selected Shahejie shales have similar variation trends across lithofacies. To further amplify the influence of $v_P(0^\circ )$ on lithofacies identification and improve the discrimination accuracy, P-wave impedance ($AI(0^\circ )$) is specially selected as the other sensitive parameter.

**Table 1:** The distribution range of vertical P- and S-wave velocities and anisotropy parameters $\epsilon$ and $\gamma$ for different lithofacies.

Samples	Argillaceous shale	Siliceous shale	Mixed shale	Calcareous shale
$v_P(0^\circ )$, km/s	(3.51, 4.11)	(3.62, 5.31)	(3.67, 5.34)	(4.81, 6.30)
$v_S(0^\circ )$, km/s	(2.21, 2.59)	(2.30, 3.11)	(2.04, 2.99)	(2.53, 3.28)
$\epsilon$	(0.15, 0.37)	(0.00, 0.37)	(0.00, 0.38)	(0.01, 0.19)
$\gamma$	(0.12, 0.25)	(0.00, 0.27)	(0.00, 0.44)	(0.01, 0.20)

### Rock-physics-driven lithofacies identification

For lacustrine shale reservoirs with complex sedimentary pattern, lithofacies identification and prediction is a fundamental step of shale play evaluation^1^. With the above analysis, two sensitive parameters ($v_P(0^\circ )/v_S(0^\circ )$ and $AI(0^\circ )$) with robust rock physics basis are selected for this purpose. This choice is motivated by their direct theoretical connection to the fundamental elastic moduli of the rock and, crucially, by their practical derivability from conventional well-logging suites (density and sonic). This provides a significant operational advantage over other common indicators (e.g., Thomsen’s $\delta$ or calibrated brittleness indices) which often require specialized data not routinely acquired in exploration settings.

**Fig. 14:**
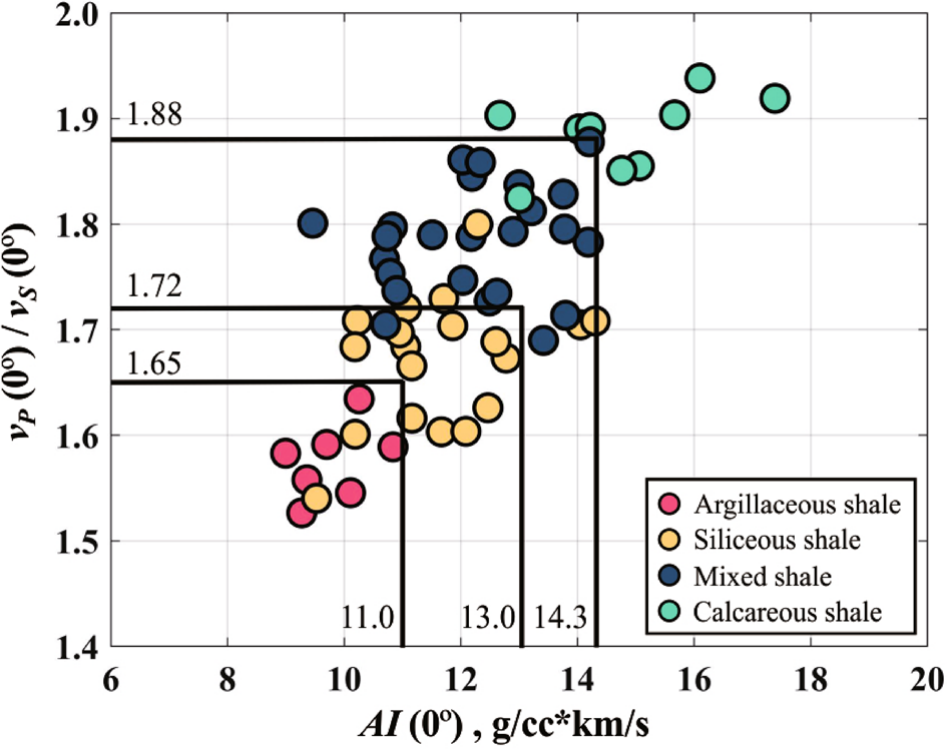
The cross-plot of $AI(0^\circ )$ versus $v_P(0^\circ )/v_S(0^\circ )$ for different lithofacies, the boundary is blocked in black lines.

Figure 14 plots $v_P(0^\circ )/v_S(0^\circ )$ as a function of $AI(0^\circ )$ for four types of lithofacies. In general, there is a positive correlation between $AI(0^\circ )$ and $v_P(0^\circ )/v_S(0^\circ )$ for all Shahejie shales, although differences exist across lithofacies. Argillaceous shales exhibit the lowest $AI(0^\circ )$ and $v_P(0^\circ )/v_S(0^\circ )$, clustering in the lower-left quadrant. $AI(0^\circ )$ and $v_P(0^\circ )/v_S(0^\circ )$ of siliceous and mixed shales are obviously higher than those of argillaceous shales. Calcareous shales have the highest $AI(0^\circ )$ and $v_P(0^\circ )/v_S(0^\circ )$, concentrated in the upper-right quadrant. The distributing boundary of different lithofacies is delineated by black lines in Fig. 14 and summarized in Table 2. Scattered data points outside these thresholds may result from localized variations in microscale mineral distribution, pore structure, or cementation heterogeneity.

Lithofacies are closely related to the quality of shale reservoirs and play a significant role in controlling oil and gas enrichment. Accurate lithofacies identification is not only the basis for studying shale reservoir characteristics, reserve calculation, and geological modeling but also a key role in reservoir quality evaluation and drilling risk analysis^38,39^. The proposed rock-physics-driven $AI(0^\circ )-v_P(0^\circ )/v_S(0^\circ )$ template, to some extent, provides a rapid way for lithofacies identification for Shahejie shale reservoir. However, the current linear models and threshold-based templates have obvious limitations. First, geological lithofacies classification requires comprehensive multi-dimensional indicators such as color, structure, TOC, and mineral compositions^40^, leading to excessive types, but the number of samples is limited, making it difficult to cover all geological types. Second, because of physical scale, core plug tests fail in providing information related to rock structure. Third, the models and templates are regional, and their applicability in other basins remains unvalidated. In the future, massive well logging data and artificial intelligence technology could be used in lithofacies prediction to increase prediction types and improve accuracy^41^. Meanwhile, upscaling analysis through numerical simulation technology may give effective prediction of structures^42^.

**Table 2:** The thresholds of P-wave impedance and velocity ratio for lithofacies classification.

Samples	Argillaceous shale	Siliceous shale	Mixed shale	Calcareous shale
$v_P(0^\circ ) / v_S(0^\circ )$	( - ,1.65)	(1.65,1.72)	(1.72,1.88)	(1.88, - )
$AI(0^\circ )$, km/s $\cdot$ $\mathrm{g/cm}^3$	( - ,11.0)	(11.0,13.0)	(13.0,14.3)	(14.3, - )

### Limitations

The laboratory-derived relationships presented herein are subject to certain constraints that should be considered for field application. The dataset is derived from a limited set of intact, centimeter-scale plug samples. Measurements conducted on these dried samples under fixed pressure provide the intrinsic elastic response of the rock matrix but may not fully replicate the in-situ behavior of fluid-saturated shales under anisotropic tectonic stress^24,43^. Furthermore, the use of high-frequency (1 MHz) ultrasonic waves introduces a scale-dependent measurement, while the relative trends between lithofacies are robust, a frequency dispersion effect is acknowledged when relating these results to lower-frequency sonic or seismic data^44,45^. Consequently, upscaling the core-plug scale elasticity and anisotropy to field seismic applications necessitates integrating additional geological heterogeneities, such as natural cracks and fractures^46^, which are beyond the resolution of this controlled experiment.

## Conclusion

In this study, we perform a suite of anisotropic velocity measurements on 59 lacustrine shale samples from Shahejie Formation, which classified into four lithofacies, argillaceous, mixed, siliceous, and calcareous, based on quantitative mineralogical thresholds. Experimental measurements reveal significant lithofacies-dependent variations in rock physics properties. The observed ultrasonic velocity anisotropy is primarily governed by the preferred orientation of clay particles, the bedding-parallel alignment of kerogen, and the associated micro-crack networks inherent to the laminated fabric of these shales.

Analysis of the velocity data establishes several key relationships with implications for reservoir characterization. A robust, globally linear trend exists between P- and S-wave velocities for all lithofacies, despite variations in their absolute $v_P-v_S$ ratios. Furthermore, the Thomsen anisotropy parameters $\epsilon$ and $\gamma$ exhibit clear exponential correlations with bedding-normal P- and S-wave velocities, respectively, offering a practical method for estimating anisotropy from simpler directional velocity logs. Ultimately, rock physics analysis identifies the bedding-normal $v_P-v_S$ ratio and P-wave impedance as the sensitive elastic parameters for discriminating between the defined lithofacies. Based on this finding, we propose a $v_P/v_S$ -versus-impedance cross-plot template with diagnostic thresholds, providing a direct application for lithofacies identification from field data.

## Data Availability

All experimental data used in this study are clearly presented within the manuscript.
